# Lymphoepithelioma epidermoid carcinoma of the uterine cervix: surgical management of an isolated case and review of the literature

**DOI:** 10.3332/ecancer.2025.1974

**Published:** 2025-08-21

**Authors:** José Richard Tenazoa-Villalobos, Edgar Fermín Yan-Quiroz, Augusto Ordoñez-Chinguel, Sofia Leonor Prado-Cucho, Vladimir Villoslada-Terrones

**Affiliations:** 1 Oncological Surgery Area of the Víctor Lazarte Echegaray Hospital – EsSalud, Trujillo 13013, Peru; 2 Faculty of Medicine, Antenor Orrego Private University, Trujillo 13008, Peru; 3 Oncological Surgery Area of the Virgen de la Puerta High Complexity Hospital - EsSalud, La Esperanza 13013, Peru; 4 National Institute of Neoplastic Diseases, Lima 15036, Peru; 5 Faculty of Medicine, Cayetano Heredia Peruvian University, Lima 150135, Peru; a https://orcid.org/0000-0003-3622-9408; b https://orcid.org/0000-0002-9128-4760; c https://orcid.org/0000-0001-8682-4948; d https://orcid.org/0000-0003-3042-0340; e https://orcid.org/0000-0001-6877-5441

**Keywords:** uterine cervical neoplasms, squamous cell carcinoma, hysterectomy

## Abstract

Cervical cancer is the gynecological malignancy that ranks third worldwide. It consists histologically of multiple subtypes, such as squamous cell carcinoma, which is the most common (65%), then adenocarcinoma (15%) and other types such as neuroendocrine, adenosquamous and carcinosarcoma tumours, which are less common. According to the World Health Organisation, lymphoepithelioma-type carcinoma has been described as an uncommon subtype and a variant of squamous cell carcinoma of the cervix. Its pathogenesis is related to the presence of the human Epstein-Barr virus and human papillomavirus. We present the case of a woman diagnosed with squamous cell lymphoepithelioma-like carcinoma of the cervix that was comprehensively managed with radical hysterectomy alone, presenting a good response and without recurrence.

## Introduction

It is estimated that cervical cancer reached an incidence of 660,000 new cases and more than 300,000 deaths worldwide in 2022. Eighty-five percent of these cases arose in low-resource countries, where cervical cancer is the second most frequent cancer and the third in mortality [1, 2].

There are two main histological types, adenocarcinoma and squamous cell carcinoma, in almost all cases associated with persistent infection with human papillomavirus (HPV). Lymphoepithelioma-like carcinoma, according to the WHO tumour classification, is a rare subtype that is considered a variant of epidermoid carcinoma. The incidence of this malignant neoplasm is 3.3% in women with FIGO 1 cervical cáncer. The pathogenesis is associated with high-risk HPV infection; some studies suggest that Epstein-Barr virus (EBV) is probably also involved, as well as in gastric and nasopharyngeal lymphoepithelioma epidermoid carcinoma (LEC), but this is still controversial and cases have been seen in Asian women [3–8].

We present the case of a 38-year-old woman with a 2 cm cervical tumour that was treated with radical hysterectomy alone and the anatomopathological study reveals the presence of lymphoepithelioma carcinoma of the cérvix. She did not receive chemotherapy or radiotherapy and currently attends periodic check-ups without showing signs of recurrence.

## Case presentation

A 38-year-old woman from Lambayeque, Tumbes, Peru, with no medical or surgical history of importance, with a gynecological history of menarche at age 14, G5P4014, first sexual intercourse at age 17 with only one partner, which she has had until now. She manifests 5 months of illness characterised by abnormal vaginal bleeding and post coital bleeding associated with pelvic pain, lancinating type of moderate intensity not irradiated, which is why she goes to the health center of origin, where she is evaluated by a physician and the gynecological examination reveals a 2 cm tumour in the cervix, no parametrial involvement. The biopsy at the health center reveals a small cell infiltrating squamous cell carcinoma.

**Figure 1.**
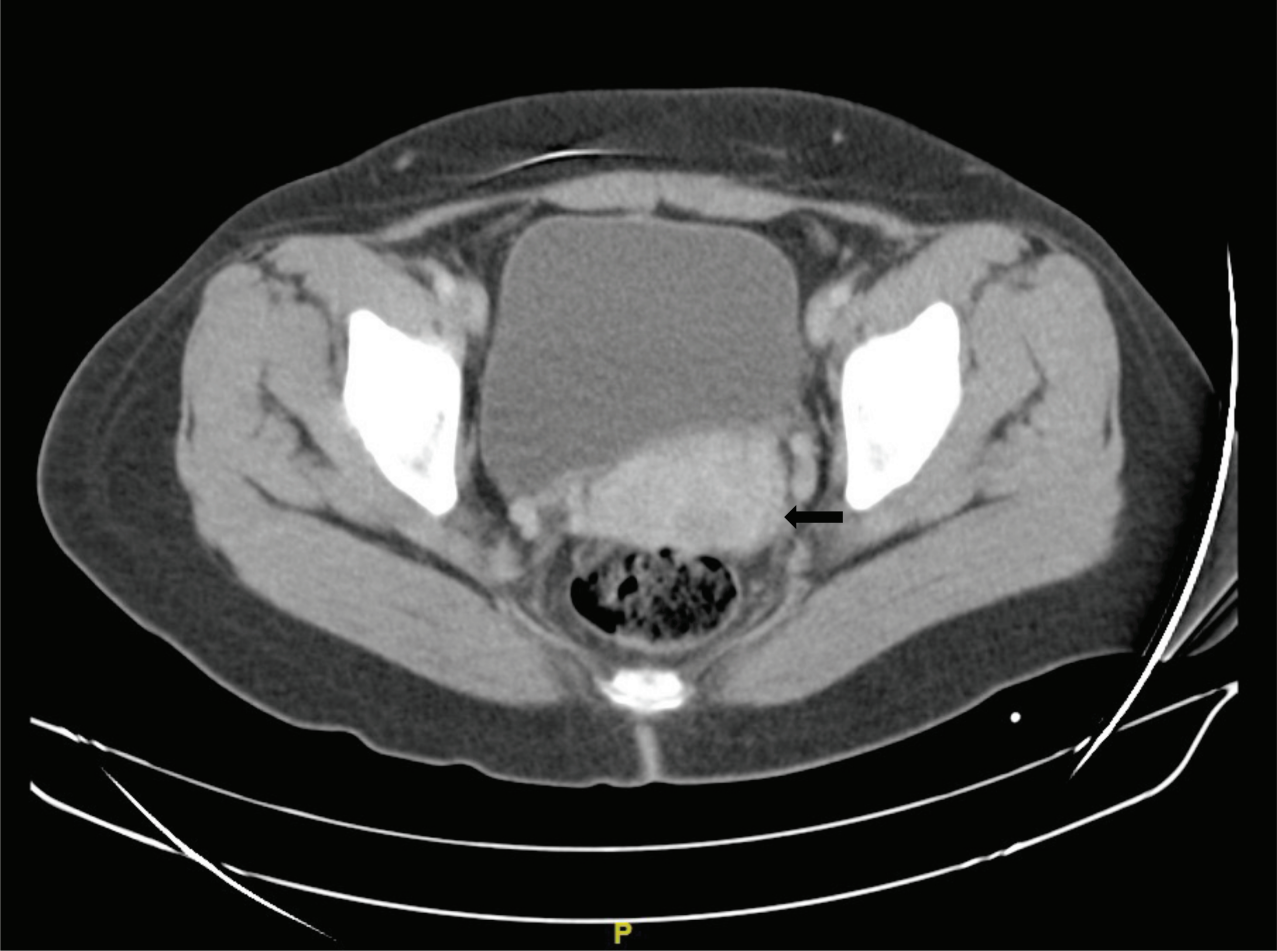
Chest, abdomen and pelvis tomography with contrast. Transverse section. Prominent uterine cervix, in the posterior lip there is a 2 × 2 cm area of contrast uptake (→).

**Figure 2.**
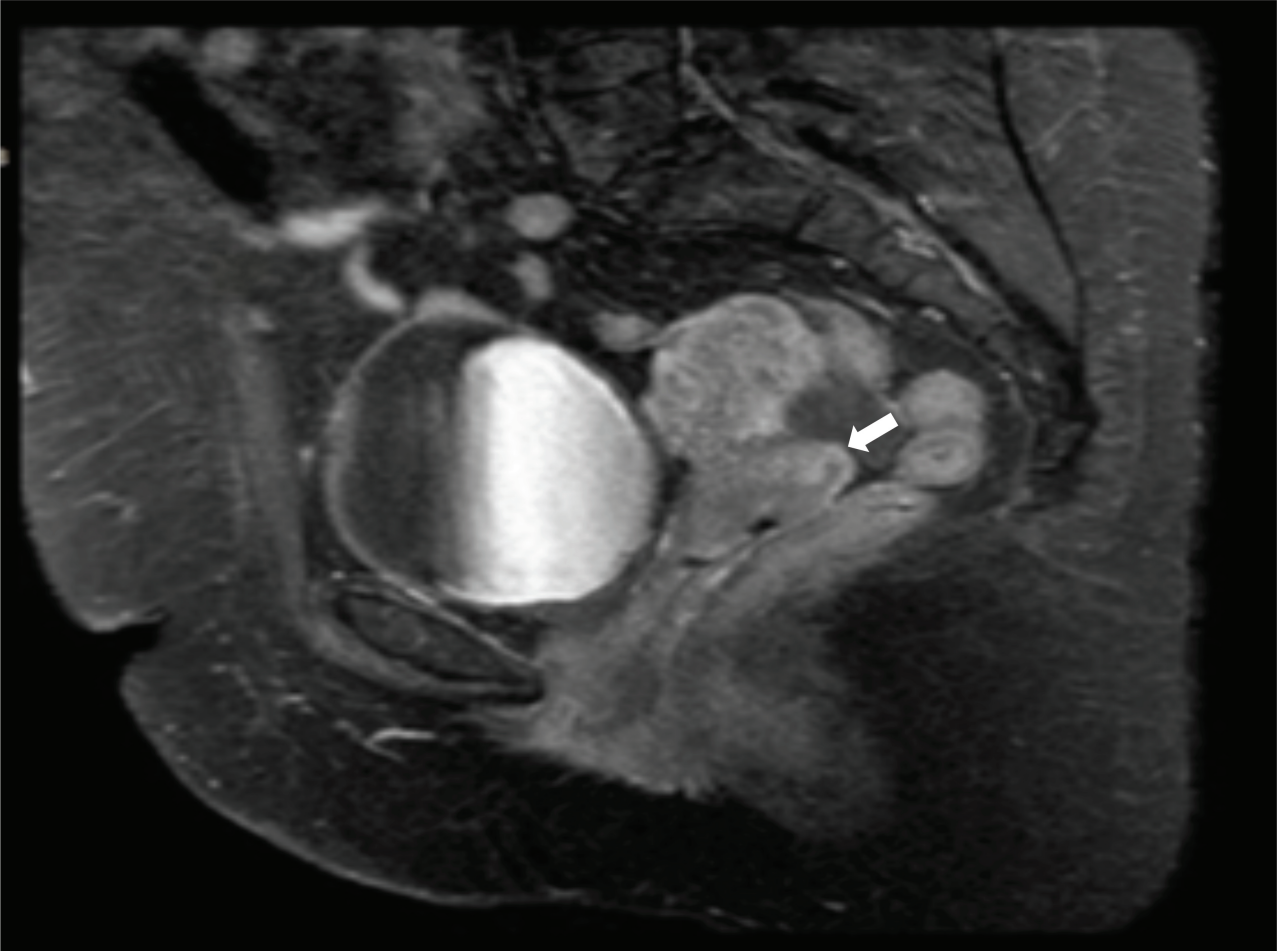
Contrast MRI of the pelvis. Sagittal view. Thickening of 2 × 2 cm at the level of the uterine cervix, predominance of the posterior lip that shows diffusion restriction and enhances with the contrast substance (→).

She is subsequently referred to the National Institute of Neoplastic Diseases (22.08.2016) and is seen in the gynecologic oncology service, where she is evaluated. Patient ECOG 1, no peripheral adenopathies. External genitalia preserved, at speculoscopy 2 cm tumour is observed dependent on the posterior lip of the cervix. Digitorectal examination: Parametrium free. Complementary studies with tomography of the thorax, abdomen and pelvis (30.08.2016): Uterus with regular borders and contours. Prominent cervix of 5.3 cm of greater diameter, in the posterior lip, there is a hypocaptive area of contrast in an extension of 2 cm, which could correspond to a lesion. No adenopathies are observed in the iliac chains. No free liquid is observed in the cavity (Figure 1). MRI of the pelvis with contrast (2.9.2016): thickening at the level of the cervix of 2 × 2 cm that shows restriction to diffusion and enhances with the contrast substance. No parametrial involvement (Figure 2).

**Figure 3.**
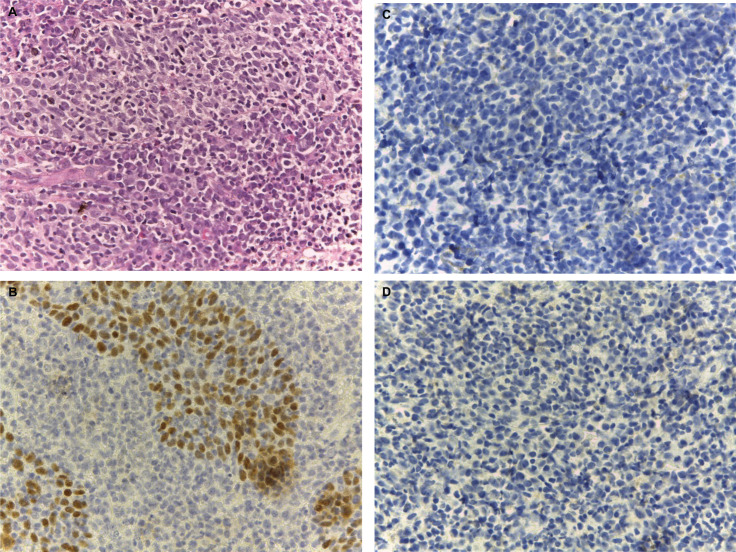
Biopsy microscopy. (a): Hematoxylin-eosin image at 40X: Malignant neoplasm composed of carcinoma component and abundant chronic inflammatory component, interspersed with nests of squamous cells. Immunohistochemistry 40X. (b): P63: Positive expression is observed in the epithelial component and negative staining in the inflammatory component. Biopsy microscopy. Immunohistochemistry 40X. (c): Synpatophysin and (d): Chromogranin, negative staining for both.

A repeat biopsy was performed, identifying a malignant neoplasm with a squamous cell component associated with severe lymphoplasmacytic inflammatory infiltrate. The squamous cell component presented nests of indistinct cell borders, scarce cytoplasm, finely granular, round or oval nuclei and prominent nucleoli, marginalised chromatin; while the inflammatory infiltrate presented predominance of lymphocytes (Figure 3A). Immunohistochemistry was performed, showing positive staining for P63 in the squamous cell component and negative staining in the inflammatory component. Also, sinpatophysin and chromogranin studies were performed, being negative for both stains (Figure 3B–D).

Patient is admitted to the operating room (14.09.2016) where Radical Hysterectomy type C1 plus oophoropexy plus bilateral pelvic lymphadenectomy is performed, the operative findings reveal a 2 × 2 cm tumour dependent on the posterior lip of the cervix, no adenopathies are observed. The patient tolerated the operative procedure, presenting a favourable evolution and was discharged on her second operative day.

**Figure 4.**
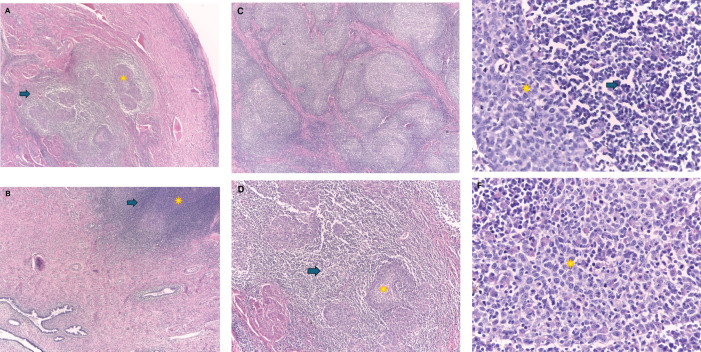
Microscopic image. Hematoxylin-Eosin-4X: In image (a): in the stroma of the uterine cervix there is proliferation of epithelioid cells (*) arranged in groups and surrounded by a dense inflammatory infiltrate (→). The exocervical epithelium does not present significant alterations. Image (b): shows few clusters of epithelioid cells with a marked inflammatory component and adjacent to these there are endocervical glands without atypia. Microscopic image. Hematoxylin-Eosin-4X: Image (c): shows nests formed by abundant inflammatory infiltrate and in the center of these groups of epithelioid cells arranged as syncytia. In image (d): at a magnification of 10X the nests are formed by epithelial cells that can form small groups (*) or be arranged in isolated cells (→). These cells present ample eosinophilic cytoplasm and the inflammatory infiltrate is predominantly formed by lymphocytes and plasmacytes. Microscopic image. Hematoxylin-Eosin-40X: Image (e): shows a nest of atypical squamous epithelial cells (*), with eosinophilic cytoplasm and indistinguishable borders, large, uniform, vesicular nuclei and prominent nucleoli, which are surrounded by a dense inflammatory infiltrate formed by lymphocytes, plasmacytes, also few eosinophils and histiocytes can be observed (→). In image (f): atypical squamous epithelial cells (*) are observed infiltrating the stroma, which would correspond to poorly differentiated infiltrating squamous epidermoid carcinoma variant Carcinoma type lymphoepithelioma.

Pathological anatomy results are obtained:

- Microscopy (Figure 4).
  - Histologic type: Nonkeratinising infiltrating epidermoid carcinoma – Lymphoepithelioma-like carcinoma: Stromata of the uterine cervix occupied by clustered epithelioid cells bordered by a dense inflammatory infiltrate. In addition, single cells and nests consisting of abundant inflammatory infiltrate such as histiocytes, eosinophils, lymphocytes and plasmacytes, epithelioid cells in the center of these clusters are arranged syncytically. Atypical squamous epithelial cells infiltrate the stroma, which would correspond to a lymphoepithelioma-like carcinoma variant.
  - Histologic grade: Poorly differentiated.
  - Infiltration: Invasion 18 mm. Extension 13 mm.
  - Absent lymphovascular invasion.
  - Free surgical margins.
  - Uterine body: Endometrium in secretory phase, myometrium without alterations.
  - Parametrium: Right and left free, 3 left intraparametrial lymph nodes are free of malignant neoplasm are identified.
  - Free right and left trunk.
  - Right (8) and left (9) pelvic lymph nodes are free of malignancy.

After these findings, a diagnosis of lymphoepithelioma of the uterine cervix carcinoma type FIGO I B1 was confirmed. Due to the clinical stage of the disease, a medical meeting was held where it was decided that, due to the early stage of the disease, the patient would remain under observation, with strict controls and would not require adjuvant chemotherapy or radiotherapy.

The patient attends periodic check-ups until the present, showing favourable evolution with no alteration in lifestyle, preserved urinary function and no signs of recurrence, metastasis or suspicious lesions.

## Discussion

Lymphoepithelioma carcinoma is an extremely rare subtype of epidermoid carcinoma of the cérvix. Microscopically, it presents similar characteristics to its counterpart in the nasopharynx and other locations. The squamous cells are undifferentiated and arranged in syncytial growth and present indistinguishable borders, the nuclei are large, monotonous and nucleolus-like with frequent mitoses, asyloid cells with poorly differentiated nuclei that are infiltrated by lymphocytes can also be found, even previously it has been designated as an inflammatory cervical neoplasm. On the other hand, the inflammatory infiltrate is dense and is composed of lymphocytes, monocytes, histiocytes and eosinophils; these inflammatory cells can present high values of CD3+ and CD8+ [6, 9].

The pathogenesis of LEC is uncertain, this neoplasm can be associated to the presence of the EBV gene, a study in Asian women has registered up to 73.3% of cases of cervical LEC had positivity for antibodies directed against EBV, in comparison with 26.7% registered in typical epidermoid carcinomas; however, there are no definitive findings that point to EBV as the main etiological factor [9–11]. Likewise, it is known that there is an association with the human papillomavirus (HPV) [16–18]. When the gene has been sequenced, an association of around 80% has been found in typical epidermoid carcinomas versus 20% found in LEC [12].

This type of neoplasm manifests in patients younger than 40 years, which is an earlier age than the other types of malignant tumours of cervical carcinoma, a 10-year study in one institution evaluated 630 patients, 17 of which were cervical LEC (3.3% of all cases in FIGO stage I), all patients were directed to surgery and none had neoadjuvant therapy, the mean age was 49.6 years (range 32–67 years). Also, in all women, radical hysterectomy was performed, plus complete pelvic lymph node dissection after that received adjuvant radiotherapy. 47.1% tested positive for HPV and EBV staining. The FIGO IB1 stage was found in 82.4%, followed by 17% as FIGO IB2, 76% had no lymph node metastasis and 17% had positive lymph nodes. It should also be noted that the incidence is much higher in Asia than in other countries such as Latin America, and the overall 5-year survival rate for this neoplasm is around 76.4%. Our patient was 38 years old at the time of diagnosis, which is younger than the mean age described, with a stage and management similar to that described, providing excellent survival [13–15].

Although the histologic features of cervical LEC presume aggressiveness, the prognosis is, on the contrary, better than any other type of squamous cell carcinoma of the uterine cervix. Compared to other cancers at the same FIGO stage, LEC is distinguished by a very low metastasis to regional lymph nodes. As in other organs, LEC has been associated with favourable patient outcomes. It is believed that the good prognosis is associated with both cellular and humoral immune reactions induced by the tumour, and it is even suggested that the immune reaction is associated with the response to antigens produced by the tumour. The lymphocytic infiltrate that lodges in the stroma reflects the humoral and cellular response against the tumours. This reaction reduces metastasis to regional lymph nodes and increases overall survival. In the case of our patient, there was no lymphovascular or perineural invasion, free surgical margins and, above all, no lymph node or distant metastasis [9–16].

The biopsy on two occasions revealed an epidermoid carcinoma of the cervix. It should be noted that when a sample is obtained from an inflammatory lesion, it is complex to make an accurate diagnosis; therefore, a complete analysis of an operative specimen from a hysterectomy is required for a definitive diagnosis. LEC is an important differential diagnosis and confusion with other types that carry a poor prognosis should be avoided. In our patient, the initial biopsies showed an epidermoid carcinoma and the diagnosis was confirmed after performing a radical hysterectomy, which is the surgical treatment of choice, and we also preserved the ovaries to avoid early menopause [17].

LEC of the uterine cervix is a rare entity; more knowledge is needed to determine the appropriate treatment for this type of neoplasm. The patient has been closely monitored since then, and no signs of recurrence have been observed. While radiotherapy has specific indications at any stage of the disease, surgery is the management of choice for early stages and LECs have better survival than other histological types of cervical cancer [6]. One study shows that cervical LECs have a lower frequency of recurrence and nodal metastasis [18]. Table 1 lists the cases of SCC managed surgically with good results.

Radical hysterectomy is the management used to treat cervical LEC. In our case, Radical Hysterectomy type C1 with oophoropexy and bilateral pelvic lymphadenectomy was performed. Surgery is planned based on imaging findings, preferably pelvic MRI. Lymph node freezing or sentinel lymph node biopsy is not performed in this case because indocyanine green is not available in our institution, and this procedure is still being validated, so it is not a standard treatment yet, and it is being performed in isolation. Oophoropexy is an acceptable management for the patient due to the age of presentation, thus avoiding early menopause and the wave of symptoms that this entails. Considering that cervical LEC is an infrequent neoplasm, many elements remain to be taken into account, so further research is needed to understand the optimal treatment for cervical LEC.

In our case, there is evidence of safe management by radical hysterectomy for LEC of the cervix, being notorious that the patient did not need chemotherapy or radiotherapy and to date shows no signs of recurrence, nodal or distant metastasis.

## Conclusion

LEC is an extremely rare entity that, despite the morphological characteristics that appear aggressive, has a favourable prognosis in relation to other types of squamous cervical cancer. It should be included in the differential and thus provide adequate management. Due to the rarity of this neoplasm, some aspects are unknown, so it is necessary to carry out extensive studies in this field.

## Conflicts of interest

The authors declare that they have no conflicts of interest associated with the manuscript.

## Funding

The case presented was entirely self-financed by the authors and did not receive any external funding.

## Ethical aspects

The written informed consent was approved by the patient for the purpose of publication of the case presented, as well as the attached images. We have the approval of the Ethics Committee of the National Institute of Neoplastic Diseases.

## Author contributions

JRTV Y EFYQ Preparation, creation and presentation of the paper, drafting of the manuscript and supervision. AOC & SPC Provision of anatomic pathology materials and drafting of manuscript. JRTV, EFYQ and VVT drafting of manuscript. JRTV & EFYQ Approval of manuscript submission.

## Figures and Tables

**Table 1.** Described cases of epidermoid cervical cancer of the lymphoepithelioma type reported in Pubmed managed with surgery and optimal survival.

Author	Year	Patient age (Years)	FIGO Stadium	Treatment	Survival
Tenazoa-Villalobos JR, Yan-Quiroz EF, Ordoñez-Chinguel A Prado-Cucho SL, Villoslada-Terrones, V.	2024	38	IB1	Surgery: Radical hysterectomy type C1 plus oophoropexy plus bilateral pelvic lymphadenectomy in 2016. Chemotherapy: No Radiotherapy: No	Patient currently alive, 8 years of follow-up and shows no signs of recurrence, metastasis or other complications.
Miyama Y, Kato T, Sato M, Yabuno A, Hasegawa K, Yasuda M [17].	2024	34	IB2	Surgery: cervical conization followed by radical hysterectomy plus bilateral pelvic lymphatic dissection. Chemotherapy: No Radiotherapy: No	Patient currently alive, no signs of recurrence during follow-up.
Kim SH, Yoon HJ, Lee NK, Choi KU, Kim KH, Suh DS [19].	2022	28	IB1	Surgery: conservative fertility surgery, open radical trachelectomy plus bilateral pelvic lymphadenectomy, plus two margin extensions, endocervical curettage and loop electrosurgical excision for resection of the endocervix followed by abdominal cerclage. Chemotherapy: Yes, adjuvant with 6 cycles of cisplatin. Radiotherapy: No	Patient follow-up for 1 year with no evidence of recurrence or other complications.
Yun HS, Lee SK, Yoon G, Kim HG, Lee DH, Na YJ, Choi OH, Shin DH, Song YJ [10]. ^.^	2015	45	IB1	Surgery: radical hysterectomy and bilateral pelvic lymph node dissection. Chemotherapy: No Radiotherapy: No	Follow-up for 8 months without adjuvant treatment since surgery, no evidence of tumor recurrence or metastasis.
Hsu LC, Lin JC, Ho SP [20].	2016	68	IB1	Surgery: two previous cervical conizations. And then radical hysterectomy plus bilateral salpingo-oophorectomy plus lymphatics and para-aortic lymph node sampling. Chemotherapy: No Radiotherapy: No	Patient has been followed for 18 months with no evidence of disease recurrence or side effects.
Takebayashi K, Nishida M, Matsumoto H, Nasu K, Narahara H [21].	2015	45	IB2	Surgery: radical hysterectomy Chemotherapy: No Radiotherapy: No	Follow-up for 4 months, no evidence of recurrence.
Saroha V, Gupta P, Singh M, Dhingra K, Khurana N [16].	2010	40	IB1	Surgery: radical hysterectomy plus ovarian conservation and pelvic and para-aortic lymphatic dissection. Chemotherapy: No Radiotherapy: No	Follow-up for 14 months, no signs of recurrence.
		43	IB1	Surgery: radical hysterectomy plus bilateral salpingo-oopherectomy and pelvic and para-aortic lymphatic dissection. Chemotherapy: No Radiotherapy: No	Follow-up for 1 year, no signs of recurrence
Kaul R, Gupta N, Sharma J, Gupta S [22].	2009	42	I	Surgery: radical hysterectomy and pelvic lymph node dissection Chemotherapy: No Radiotherapy: No	Disease-free 12 months after diagnosis.
El Hossini Soua A, Trabelsi A, Laarif M, Mutijima E, Sriha B, Mokni M, Korbi S [23].	2004	79	I	Surgery: radical hysterectomy Chemotherapy: No Radiotherapy: No	Follow-up for 6 months, no signs of recurrence.
Saylam K, Anaf V, Fayt I, Noel JC [24].^.^	2002	72	IB	Surgery: radical hysterectomy plus bilateral pelvic lymphadenectomy. Chemotherapy: No Radiotherapy: No	Not specified
López-Ríos F, Miguel PS, Bellas C, Ballestín C, Hernández L [12].	2000	44	IB2	Surgery: radical hysterectomy plus bilateral pelvic lymphadenectomy. Chemotherapy: No Radiotherapy: No	Follow-up for 12 months, disease-free
Pires MA, Andrade MJ, Guerra C, Silva TS, de Oliveira C, Beja M [25]	1999	33	IIB	Surgery: radical hysterectomy plus bilateral pelvic lymphadenectomy. Chemotherapy: Yes Radiotherapy: Yes, due to right and left internal iliac lymph node involvement, obturator and parametrial fossa.	Patient traveled to Germany, where he remains in controls without signs of recurrence.
